# Influence of Psychological Nursing Procedure on Negative Emotion, Stress State, Quality of Life and Nursing Satisfaction in Patients with Lung Cancer Radical Operation

**DOI:** 10.3389/fsurg.2022.899033

**Published:** 2022-05-04

**Authors:** Yizhi Yu, You Xia, Xiaoyan Fan, Yong Chen, Chengjuan Li, Jing Zhang

**Affiliations:** ^1^ Nursing Department, The First Hospital of Changsha, Changsha City, China; ^2^ Internet Hospital Office, The First Hospital of Changsha, Changsha City, China; ^3^ Early Clinical Research Center, Hunan Cancer Hospital, Changsha City, China; ^4^ School of Nursing, University of South China, Hengyang City, China; ^5^ Second Department of Thoracic Medicine, Hunan Cancer Hospital, Changsha City, China

**Keywords:** lung cancer radical operation, psychological nursing procedure, negative emotion, stress state, quality of life, nursing satisfaction

## Abstract

**Objective:**

To discuss the influence of psychological nursing procedure on negative emotion, stress state, quality of life and nursing satisfaction in patients with lung cancer radical operation.

**Methods:**

106 patients with lung cancer who underwent radical resection in our hospital from September 2019 to September 2021 were selected. According to the intervention time, patients were divided into Group A and Group B, with 53 cases in each group. Group A received routine nursing, Group B used psychological nursing procedure on the basis of Group A. The negative emotions, stress state, quality of life and nursing satisfaction of patient were observed.

**Results:**

Self-rating anxiety scale and self-rating depression scale scores of Group B were lower than Group A (*P* < 0.05). The levels of norepinephrine, epinephrine and cortisol in Group B were lower than Group A (*P* < 0.05). Generic quality of life inventory-74 scores of Group B were higher than Group A (*P* < 0.05). The nursing satisfaction of Group B (88.68%) was higher than Group A (73.58%) (*P* < 0.05).

**Conclusion:**

Psychological nursing procedure is conducive to reducing the negative emotion, relieving stress reaction, improving the quality of life, increasing nursing satisfaction of patients with lung cancer radical operation.

## Introduction

With the deterioration of the environment and the increase of people’s unhealthy living habits, the incidence of lung cancer has risen sharply. Because the symptoms of lung cancer in the early stage are concealed and lack of specific clinical symptoms, most of them are common symptoms, such as cough and chest tightness, so it is difficult to attract the attention of patients (1). By the time a patient is diagnosed with lung cancer, the disease is often in the middle and late stage, and the 5-year survival rate of the patient is only 10%–15% (2). It is reported that in 2020, the most common cancer in China is lung cancer (820,000), and the death toll is far ahead, reaching 715,000 (3). In recent years, with the continuous development of medical technology, lung cancer resection has become the most effective method for the treatment of lung cancer. Surgery has improved the survival rate of patients with lung cancer to a certain extent, and brought hope to countless people suffering from cancer (4). However, after surgical resection of the lesion, the patient will have some trauma, and the the lung function of the patient will be damaged, thus triggering the body’s stress response. Long-term severe stress will promote the abnormal release of histamine, catecholamine and other substances, and then affect the physical function (5, 6). At the same time, surgery, postoperative chemotherapy, pain of malignant tumor themselves and complications will cause double burdens on the patients’ physiology and psychology, resulting in negative psychology such as irritability, anxiety and depression, which will affect the treatment effect of lung cancer and reduce the quality of life of patients (7). Therefore, it is very urgent to actively explore and improve the psychological status of patients during treatment. Psychological nursing procedure is a nursing method guided by psychological theory. It deals with various psychological problems by observing the law of patients’ psychological activities, and then changes the patient’s behavior and improves the outcome of the disease. We observed the changes of patients’ negative emotions, stress state, quality of life and nursing satisfaction before and after the implementation of psychological nursing procedure for patients with lung cancer radical operation.

## Materials and Methods

### Research Object

106 patients with lung cancer who underwent radical resection in our hospital from September 2019 to September 2021 were selected as the research object. Inclusion criteria: ① meeting the diagnostic criteria of lung cancer (8); ② There are indications for radical resection of lung cancer and no surgical contraindications; ③ No radiotherapy or chemotherapy before operation; ④ The expected survival time was >3 months; ⑤Cognitive, communication and writing skills are barrier-free. Exclusion criteria: ① suffering from autoimmune diseases; ② Complicated with functional diseases of important organs; ③ Other malignant tumors; ④ Complicated mental illness; ⑤ Have received similar mental health treatment in the past; ⑥ The patient was not the first to undergo surgery. According to the intervention time, patients were divided into Group A (from September 2019 to September 2020) and Group B (from September 2020 to September 2021), with 53 cases in each group.

### Research Methods

Group A received routine nursing. Including: completed the health education work, introduced the current condition to patients, explained the operation plan, operation precautions and possible complications. Explained to patients the importance of maintaining good psychology, informed and supervised patients to quit smoking and drinking absolutely, and implement diet guidance.

Group B used psychological nursing procedure on the basis of Group A. ① Psychological nursing teams were established. All members team members were required to have at least 3 years of clinical work experience, and they must received training on psychological nursing related knowledge before joining the group. Only those who pass the examination can be enrolled into the group. The training content included the knowledge related to lung cancer disease, nursing measures and coping measures related to cancer radiotherapy and chemotherapy. ② Team members assessed the psychological status of patients, fully communicated with patients and established a good doctor-patient relationship. Through the results of psychological assessment to grasp the causes of patients’ unhealthy psychology, and formulated corresponding psychological intervention programs. Focused on observing patients’ psychological changes, communicated with patients who have negative emotions as soon as possible, and eased their unhealthy psychology. Patients were guided to establish self-management and psychological adjustment ability, explained the adverse effects of bad psychology to patients, and informed patients to keep a good state of mind. ③ The team members gave health education manuals to patients and their families, and taught the patients about lung cancer related knowledge, related effects of radiotherapy, adverse reactions of operation, etc., so as to help the patients understand their own diseases and relieve their fear. ④ The society and family were encouraged to provide emotional support to patients, and group activities such as communication meetings and outdoor outing were regularly organized, so that patients can realize their own value. Patients were encouraged to maintain communication with the outside world, so that patients could feel the meaning of life. Patient’s family was instructed to spend more time with the patient, so that the patient could establish a positive and optimistic attitude and reduce negative emotions. ⑤ A supervisory group was established, consisting of head nurses and psychological counselors, and the supervisory group was supposed to oversee the implementation of the entire nursing process and the quality of care.

### Observation Index

① Medical records were established for all patients when they were admitted to hospital, and personal information, disease information and other information were collected. ② Self-rating anxiety scale (SAS) and self-rating depression scale (SDS) were used to evaluate patients’ negative emotions. The contents of SAS and SDS include 20 items, each of which was divided into 4 grades, and the demarcation values of standard scores were 50 points and 53 points respectively. The total score of SAS and SDS ranges from 20–80 points. The degree of anxiety and depression was directly proportional to the score. ③ The fasting venous blood of patients was collected, after centrifugation, the levels of norepinephrine (NE), epinephrine (E) and cortisol by radioimmunoassay were measured. ④ Generic quality of life inventory-74 (GQOLI-74) was used to evaluate the patients’ quality of life. Include 74 items, each of which was divided into 5 levels. They were 4 dimensions: psychological function, social function, material life and physical function. The total score for each dimension was 100 points. The quality of life is directly proportional to the score. ⑤ The self-made nursing satisfaction scale was used to evaluate patients’ satisfaction. It include nurses’ attention to patients, nurses’ communicability, professional knowledge reserve, nursing accessibility and nursing service quality. Each item was divided into 5 grades, with a total score of 100 points, with >80 points as satisfied, 60–80 points as relatively satisfied and <60 points as dissatisfied. Total satisfaction = satisfied + relatively satisfied. The Cronbach’s α of scale was 0.776. The scale has good reliability.

### Statistical Methods

SPSS 22.0 software was used for analysis, measurement data were expressed as mean ± standard deviation, *t*-test was used to analyze the comparison. Count data was expressed as a ratio, *χ*^2^-test was used to analyze the comparison. *P* < 0.05 was statistically significant.

## Results

### Patients’ Clinical Data

There was no significant difference in clinical data between the two groups (*P* > 0.05). (Table 1).

**Table 1:** Patients’ clinical data (*n*, %, $\bar{x}\pm s$).

Group	Gender		Age (years)	Pattern of organization		TNM staging		
	Male	Female		Squamous carcinoma	Glandular cancer	Stage I	Stage II	Stage III
Group A (*n* = 53)	30 (56.60%)	23 (43.40%)	63.82 ± 5.66	38 (71.70%)	15 (28.30%)	21 (39.62%)	29 (54.72%)	3 (5.66%)
Group B (*n* = 53)	34 (64.15%)	19 (35.85%)	62.95 ± 5.51	36 (67.92%)	17 (32.08%)	20 (37.74%)	31 (58.49%)	2 (3.77%)
*χ*^2^*/t* value	0.631		0.802	0.179		0.291		
*P* value	0.427		0.424	0.672		0.865		

### Patients’ Negative Rmotions

**Figure 1:**
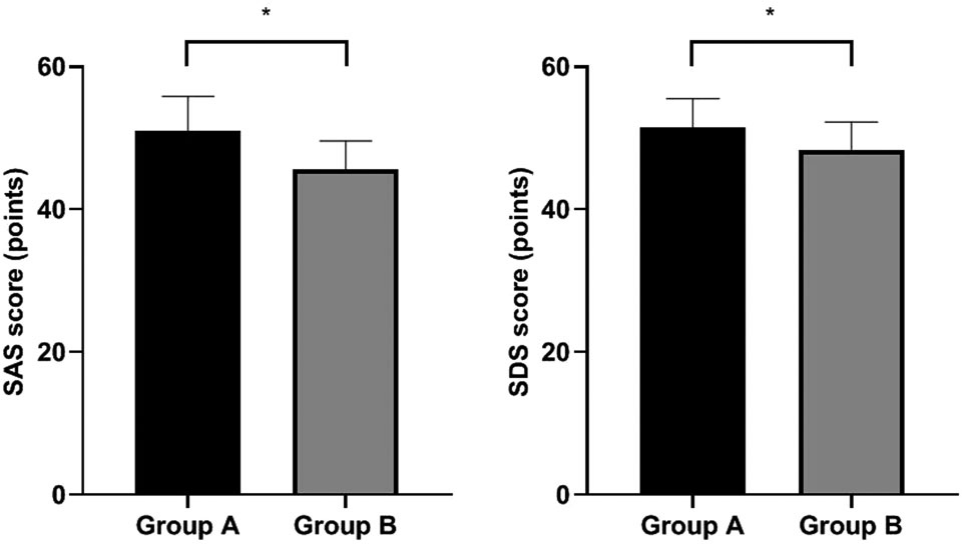
Patients’ negative emotions. Compared with Group A, *P < 0.05.

SAS and SDS scores of Group B were lower than Group A (*P* < 0.05). (Figure 1).

### Patients’ Stress State

**Figure 2:**
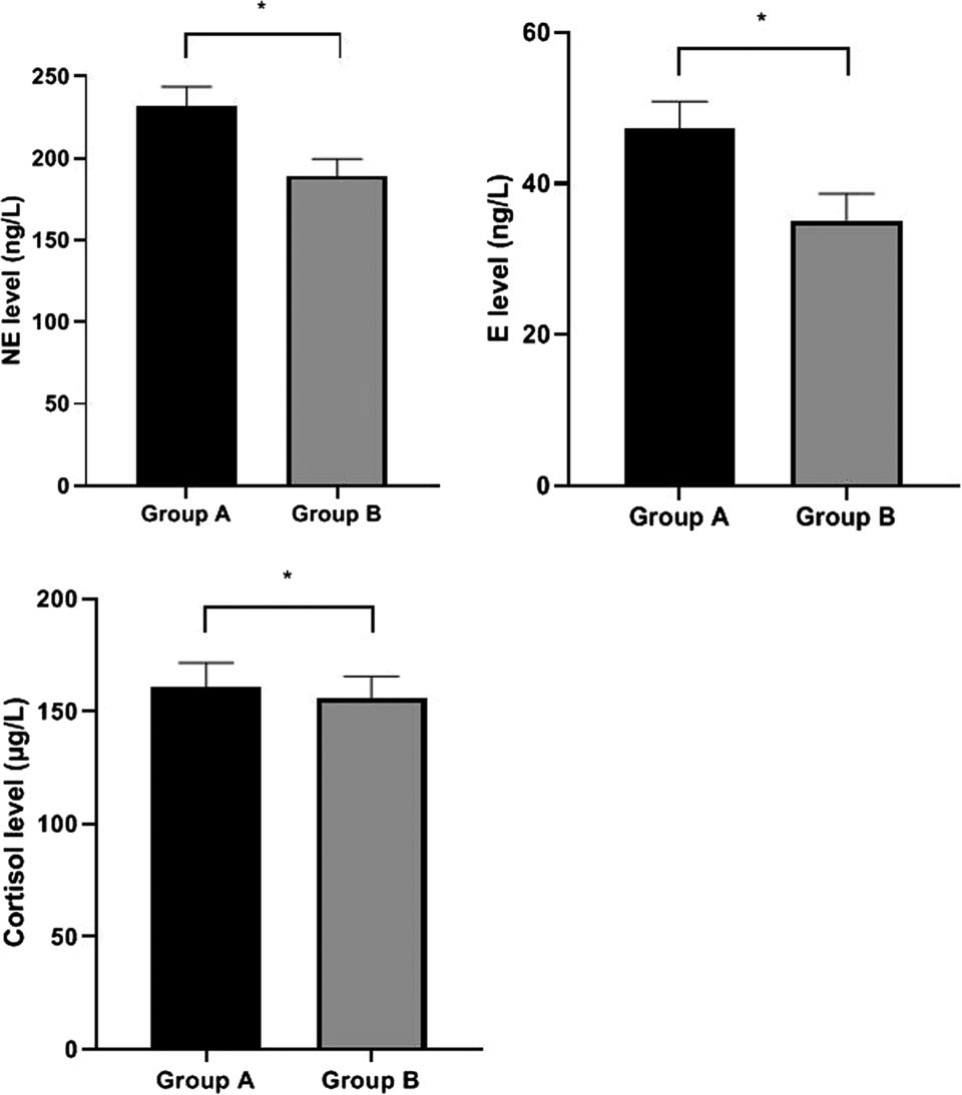
Patients’ stress state. Compared with Group A, *P < 0.05.

The levels of NE, E and cortisol in Group B were lower than Group A (*P* < 0.05). (Figure 2).

### Patients’ Quality of Life

**Figure 3:**
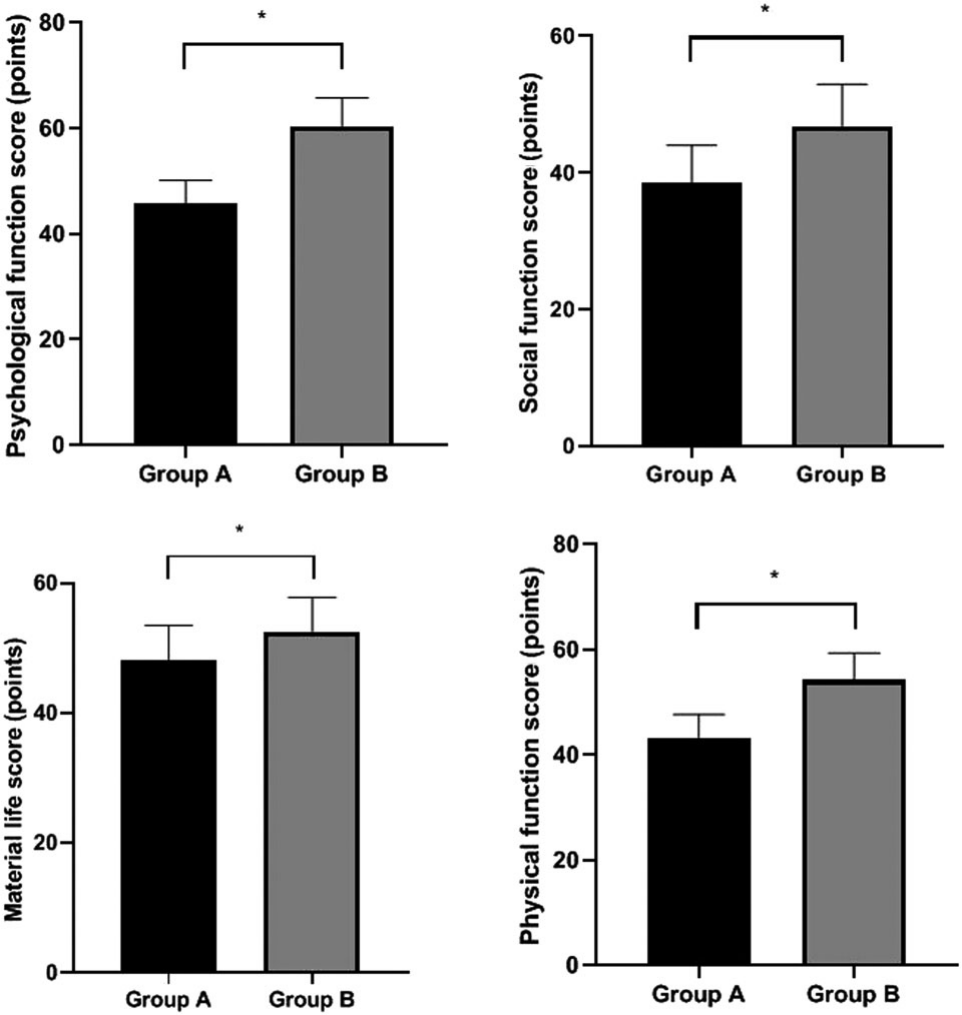
Patients’ quality of life. Compared with Group A, *P < 0.05.

GQOLI-74 scores of Group B were higher than Group A (*P* < 0.05). (Figure 3).

### Patients’ Nursing Satisfaction

The nursing satisfaction of Group B (88.68%) was higher than Group A(73.58%) (*P* < 0.05). (Table 2).

**Table 2:** Patients’ nursing satisfaction (*n*, %).

Group	Satisfied	Relatively satisfied	Dissatisfied	Total satisfaction
Group A (*n* = 53)	18 (33.96%)	21 (39.62%)	14 (26.42%)	39 (73.58%)
Group B (*n* = 53)	23 (43.40%)	24 (45.28%)	6 (11.32%)	47 (88.68%)
*χ*^2^ value				3.944
*P* value				0.047

## Discussion

At present, surgical resection is often used in clinical treatment of lung cancer in China, and the survival time of patients is gradually prolonged. However, the disease of lung cancer itself and the long-term treatment process will cause patients to have different degrees of adverse reactions. Coupled with unbearable pain and high medical costs, patients are prone to negative attitudes, which seriously affects the quality of life of patients (9). At the same time, patients with lung cancer often have a strong psychological stress reaction due to the lack of disease-related knowledge, mainly manifested as emotional anxiety, depression and somatization, etc., which will have a great impact on the treatment effect (10, 11). At present, conventional nursing lacks targeted psychological counseling, and there are still some problems such as single personnel composition and incomplete nursing programs, so it cannot effectively reduce the stress reaction of patients, resulting in poor recovery of patients’ body function after operation (12). In view of this, how to adopt effective psychological intervention methods to relieve patients’ unhealthy psychology and improve the level of hope of patients has become an important topic in the treatment of lung cancer.

Due to the lack of understanding of the disease and the uncertainty of the operation results, patients with lung cancer undergoing radical resection are prone to negative emotions. Excessive negative emotions will affect the patient’s multiple physiological systems and affect the patient’s recovery, while poor recovery will aggravate the patient’s negative emotions, thus forming a vicious circle (13, 14). We found that psychological nursing procedure for patients undergoing radical resection of lung cancer can reduce SAS and SDS scores. The psychological nursing procedure is aimed at the role of the patient, and applies the biological-psychological-social medical model to the treatment process of patients, so as to improve the patient’s psychological condition and optimize the patient’s self-emotional management ability (15). This method assesses the patient’s psychological status, master the causes of unhealthy psychology and formulate corresponding psychological intervention programs, which can help patients reduce negative psychology, alleviate the pain caused by the tumor itself and treatment, and help patients face difficulties positively (16). Cetkin’s team believed that health education is closely related to the clinical treatment effect of patients undergoing radical lung cancer surgery, but most patients lack knowledge about diseases, which often requires systematic popularization of science by medical staff (17). In the psychological nursing procedure, team members will explain the adverse effects of bad psychology on the body to the patients, and educate the patients about lung cancer-related knowledge, so as to help the patients understand their own diseases, reduce patients’ anxiety about the diseases, and relieve patients’ doubts (18). In addition, psychological nursing aims at the characteristics of psychological needs of patients undergoing radical lung cancer surgery, encourages patients to maintain communication with the outside world, enables patients to obtain social and family support, helps patients to eliminate psychological burden, increases their initiative to participate in disease treatment, and achieves the realization of psychological barriers (19).

As a stressor, surgery can excite the hypothalamus-pituitary-adrenal cortex system and locus coeruleus-sympathetic nerve-adrenal medulla system of patients, which can increase the secretion of catecholamine in the blood and cause patients to have psychological stress reaction (20). NE, E and cortisol are stress hormones secreted in large quantities by negative feedback of central nervous system under stress. When patients have strong stress reaction, the levels of NE, E and cortisol in the blood can be increased several times, and the degree of increase is positively correlated with the intensity of stress reaction (21, 22). In this study, the levels of NE, E and cortisol in Group B are lower than those in Group A. This showed that psychological nursing procedure can alleviate the stress reaction of patients. Psychological nursing procedure is patient-centered, and reasonable psychological intervention plan is formulated according to its clinical characteristics. After implementing psychological counseling, health education, emotional support and other measures for patients, it is beneficial to improve patients’ psychological resistance to face various problems and stimuli, so that they can still maintain a calm and good attitude in the face of stress, which is helpful to improve the mood of patients during treatment (23). At the same time, psychological intervention can make patients more aware of the disease and the operation process, reduce the damage to the body caused by emotional fluctuations, reduce the allowable effect of NE, E and cortisol alert reaction, and reduce the synthesis and release of endogenous opioid peptides in the brain, thereby alleviating the stress state (24). In addition, the research of van de Wiel’s team and Long’s team showed that psychological nursing can improve the quality of life and nursing satisfaction of patients undergoing radical lung cancer surgery (25, 26). It is basically consistent with the results of this study. Psychological nursing procedure can fully mobilize the subjective initiative of patients, improve the physical and psychological functions of patients, meet the needs of patients for knowledge related to diseases and operations, and finally achieve the purpose of improving patients’ quality of life, and the nursing satisfaction will also increase accordingly.

## Conclusion

To sum up, the psychological nursing procedure is conducive to reducing the negative emotion, relieving stress reaction, improving the quality of life, increasing nursing satisfaction of patients with lung cancer radical operation. This study only observed the physical and mental conditions of patients with lung cancer radical operation during the perioperative period, and it is necessary to further explore the long-term psychological state, long-term quality of life and long-term survival rate of patients in subsequent studies.

## Data Availability

The original contributions presented in the study are included in the article/supplementary material, further inquiries can be directed to the corresponding author/s.
